# Comparison of Monoamine Oxidase-A, Aβ Plaques, Tau, and Translocator Protein Levels in Postmortem Human Alzheimer’s Disease Brain

**DOI:** 10.3390/ijms241310808

**Published:** 2023-06-28

**Authors:** Amina U. Syed, Christopher Liang, Krystal K. Patel, Rommani Mondal, Vallabhi M. Kamalia, Taylor R. Moran, Shamiha T. Ahmed, Jogeshwar Mukherjee

**Affiliations:** Preclinical Imaging, Department of Radiological Sciences, University of California-Irvine, Irvine, CA 92697, USA

**Keywords:** [^18^]FAZIN3, [^18^F]flotaza, [^125^I]IPPI, [^18^F]FEPPA, human tau, Aβ plaques, Alzheimer’s disease, monoamine oxidase-A

## Abstract

Increased monoamine oxidase-A (MAO-A) activity in Alzheimer’s disease (AD) may be detrimental to the point of neurodegeneration. To assess MAO-A activity in AD, we compared four biomarkers, Aβ plaques, tau, translocator protein (TSPO), and MAO-A in postmortem AD. Radiotracers were [^18^F]FAZIN3 for MAO-A, [^18^F]flotaza and [^125^I]IBETA for Aβ plaques, [^124/125^I]IPPI for tau, and [^18^F]FEPPA for TSPO imaging. Brain sections of the anterior cingulate (AC; gray matter GM) and corpus callosum (CC; white matter WM) from cognitively normal control (CN, *n* = 6) and AD (*n* = 6) subjects were imaged using autoradiography and immunostaining. Using competition with clorgyline and (*R*)-deprenyl, the binding of [^18^F]FAZIN3 was confirmed to be selective to MAO-A levels in the AD brain sections. Increases in MAO-A, Aβ plaque, tau, and TSPO activity were found in the AD brains compared to the control brains. The [^18^F]FAZIN3 ratio in AD GM versus CN GM was 2.80, suggesting a 180% increase in MAO-A activity. Using GM-to-WM ratios of AD versus CN, a >50% increase in MAO-A activity was observed (AD/CN = 1.58). Linear positive correlations of [^18^F]FAZIN3 with [^18^F]flotaza, [^125^I]IBETA, and [^125^I]IPPI were measured and suggested an increase in MAO-A activity with increases in Aβ plaques and tau activity. Our results support the finding that MAO-A activity is elevated in the anterior cingulate cortex in AD and thus may provide a new biomarker for AD in this brain region.

## 1. Introduction

**Figure 1:**
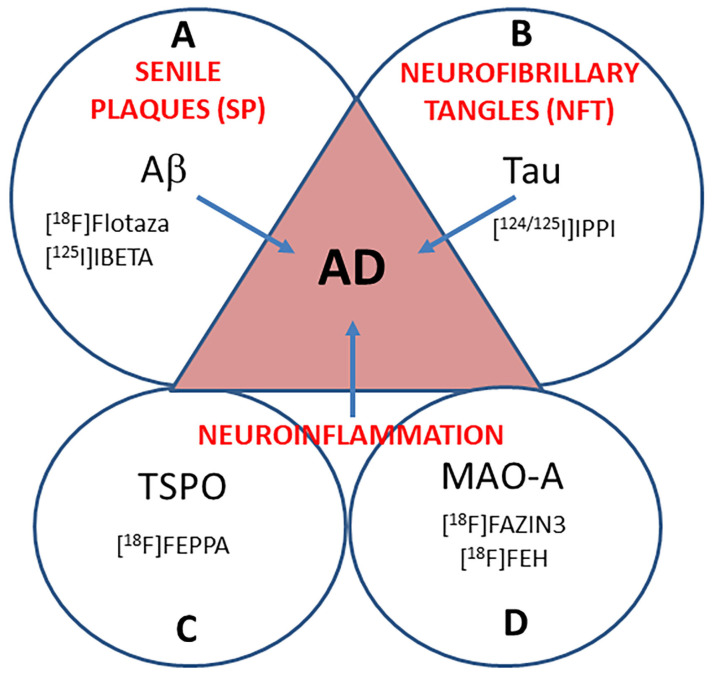
Aβ, tau, MAO-A, and TSPO in AD Subjects: Schematic showing four biomarkers for AD. (**A**). Aβ-plaque (senile plaques, SP) PET imaging agents are currently used in AD subjects. [^18^F]flotaza and [^125^I]IBETA, both Aβ plaque imaging agents, were used in this work. (**B**). Tau (neurofibrillary tangles, NFT) PET imaging agents are currently used for in AD subjects. In this work, [^124/125^I]IPPI imaging agents were used to evaluate Tau. (**C**). Translocator protein (TSPO), a biomarker for neuroinflammation, is being studied in AD using radiotracers such as [^18^F]FEPPA. (**D**). Monoamine oxidase-A (MAO-A) in AD using [^18^F]FAZIN3 as a potential new biomarker for neuroinflammation was investigated in this work.

Early diagnosis and disease monitoring strategies for Alzheimer’s disease (AD), the most common type of dementia, are critically important due to its high prevalence in an aging worldwide population. Characterized by the accumulation of amyloid β (Aβ) plaques and neurofibrillary tangles (NFT) in the brain [1,2], molecular biomarkers for AD are now indispensable for the clinical definition of the process and stage of the disease [3]. PET imaging of Aβ plaques is currently being used to monitor drug treatments [4]. There is now an increased focus on tau as a more accurate early predictive marker for AD diagnosis. Phosphorylated p-tau immunoassays p-tau181 [5] and p-tau217 [6] confirmed that plasma p-tau has high sensitivity and specificity to detect AD neuropathology, which was ascertained with [^18^F]flortaucipir PET [7,8]. Thus, both Aβ plaque and NFT PET imaging are now playing a major role in the diagnosis, staging, and treatment evaluations of AD (Figure 1).

Neuroinflammation in AD is now regarded as an early indicator of disease [9]. Translocator protein-18 kDa (TSPO) is currently being used as one such biomarker for PET imaging of neuroinflammation [10]. Alternate molecular targets of inflammation other than TSPO are now receiving attention for PET radiotracer development [11]. Monoamine oxidase-A (MAO-A), a pro-oxidative enzyme encoded by the X chromosome and located in the outer mitochondrial membrane and cytosol, has been suggested as a biomarker for activated monocytes and macrophages [12]. MAO-A plays a major role in neurotransmitter degradation in the human brain [13].

MAO-A may play a role in the regulation of neuronal survival in neurodegenerative disorders [14] as well as monoaminergic dysfunction and mitochondrial dysfunction in AD [15]. MAO-A gene polymorphisms have been shown to be associated with neurological changes in AD pathology [16]. An enzymatic assay of MAO-A activity in AD brain tissue showed an increase or no change in several brain regions [17], while another report using similar assay methods indicated a decrease in MAO-A activity in the prefrontal cortex [18]. MAO-A hyperactivity has been shown to be associated with depression, suggesting MAO-A inhibitors as effective therapeutics against clinical depression and anxiety [19]. Upregulation of MAO-A has been reported with chronic intermittent hypoxia and oxidative stress leading to neurodegeneration [20]. To the best of our knowledge, no imaging studies (autoradiographic or PET) of MAO-A in AD have been reported.

Imaging studies using PET on the isozyme MAO-B have been reported in AD. Initial studies on MAO-B as an off-target of tau imaging agents and subsequent studies have shown an increase in MAO-B activity in AD [21,22]. Recent PET imaging findings using the new reversible MAO-B [^18^F]SMBT-1 suggest an increase in MAO-B activity in the human AD temporal cortex that is concordant with the presence of tau and may suggest inflammatory changes [23]. This increased MAO-B in AD patients using PET imaging is consistent with previous studies which found increased MAO-B activity in AD [17,18].

Using autoradiographic imaging methods, we have evaluated MAO-A activity in postmortem human Parkinson’s disease (PD) brain sections of the anterior cingulate using [^18^F]FAZIN3, a new reversible MAO-A inhibitor [24]. Our ongoing multitarget approach involves comparing Aβ plaque imaging using [^18^F]flotaza [25], tau imaging using [^125^I]IPPI [26], and TSPO imaging for microglia using [^18^F]FEPPA [27] with [^18^F]FAZIN3 and [^18^F]fluoroethyl harmol ([^18^F]FEH; [28,29]) for MAO-A imaging. The human anterior cingulate (AC) cortex has been shown to be enriched with MAO-A in PET studies [30], and this region has abundant accumulation of Aβ plaques [31] and tau [32] in AD patients. Microglial activity in the anterior cingulate cortex is being investigated in brain disorders and is therefore a good biomarker to be explored [33]. Because of the presence of these biomarkers in AC and the important role AC plays in cognitive function [34], this brain region was chosen for the present study. The four biomarkers were evaluated in cognitively normal (CN) and AD subjects using autoradiography to assess potential relationships between the biomarkers in AD (Figure 1).

## 2. Results

### 2.1. MAO-A Imaging

**Figure 2:**
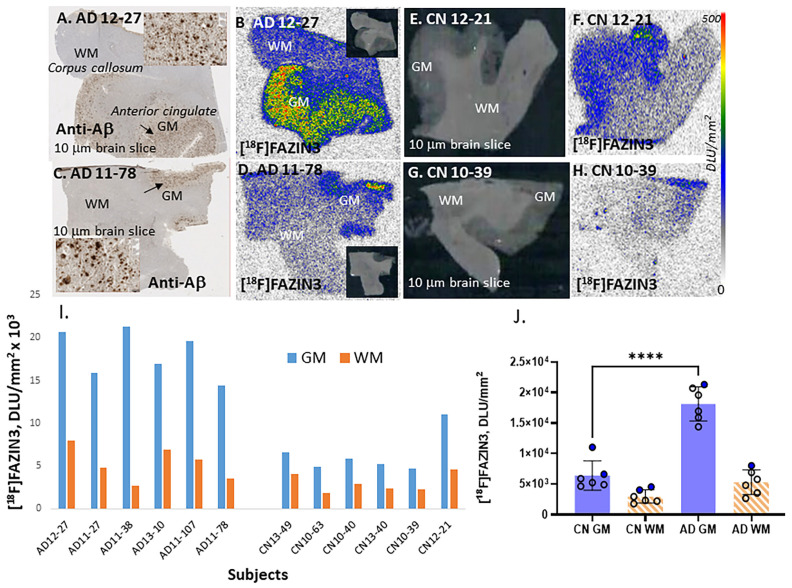
MAO-A Imaging of [^18^F]FAZIN3: (**A**,**C**). Anti-Aβ immunostained brain slices (10 μm) of two AD subjects (AD 12-27 and AD 11-78) showing abundant Aβ plaques (arrows) in the GM regions of the anterior cingulate (inset shows GM with Aβ plaques). (**B**,**D**). Binding of [^18^F]FAZIN3 in AD 12-27 and AD 11-78 brain slice GM is significantly greater compared to the lower levels in corpus callosum, WM. (**E**,**G**). Brain slices (10 μm) of CN subjects (CN 12-21 and CN 10-39) show GM regions of anterior cingulate, (**F**,**H**). Binding of [^18^F]FAZIN3 in the CN brain GM and lower levels WM. (**I**). Plot of GM and WM of all AD and CN subjects. [^18^F]FAZIN3 binding is seen in all CN and AD subjects, with GM showing higher levels compared to WM. AD brains show greater [^18^F]FAZIN3 in GM regions compared to control subjects. (**J**). Plot shows averages of all AD and CN subjects (“**** = *p* < 0.0001” for AD GM versus CN GM, unpaired two-tailed *t*-test and *p* < 0.05 for AD WM versus CN WM; open circles males, solid circles females). Ratio of AD GM/WM = 3.43 whereas CN GM/WM = 2.17. Autoradiography scale bar: 0–500 digital light units (DLU)/mm^2^.

[^18^F]FAZIN3 is a polyethyleneglycol (PEG) fluoroalkyl azaindole derivative and binds reversibly and selectively to MAO-A [24]. Figure 2 shows images of two AD subjects and two CN subjects from the list in Table 1. Anti-Aβ IHC of the two AD subjects are shown in Figure 2A,C, confirming the presence of abundant Aβ plaques in the gray matter regions of the anterior cingulate. Greater binding of [^18^F]FAZIN3 was distinctly seen in the anterior cingulate in both AD subjects (Figure 2B,D), with lower levels in the WM regions.

The average ratio of GM/WM in the six AD subjects was 3.43. Two CN subjects shown in Figure 2E,G also showed more [^18^F]FAZIN3 binding in the GM compared to that in the WM (Figure 2F,H). This is consistent with the presence of MAO-A in the human anterior cingulate [30]. The ratio of GM/WM in the six CN subjects averaged 2.17. None of the CN subjects except for CN12-21 (Figure 2E,F) exhibited a presence of Aβ plaques (Table 1), and this was confirmed using [^18^F]flotaza and [^125^I]IBETA [35]. The binding of [^18^F]FAZIN3 in the GM and WM of all the AD and CN subjects is shown in Figure 2I, and the average of the GM and WM in the two groups of subjects is also shown in Figure 2J. The average [^18^F]FAZIN3 AD GM-to-CN GM ratio = 2.80, suggesting a significant increase in [^18^F]FAZIN3 in AD subjects (*p* < 0.001). When comparing the GM/WM ratios of AD (3.43) to those of CN (1.58), a 58% increase in [^18^F]FAZIN3 binding was observed, suggesting an increase in MAO-A activity.

**Table 1:** Patient samples and data *.

ID	CERAD Pathology	Gender	Age	PMI, hrs	Brain Region ^1^	Plaque Total	Tangle Total	LB	Braak Score
10-39	CN	Male	93	3	AC	0	1	0	I
10-63	CN	Male	79	3	AC	0	2.5	0	II
10-70	CN	Male	74	3.25	AC	0	2	0	I
12-21	CN ^2^	Female	88	1.25	AC	14	3.5	0	II
13-40	CN	Male	73	4.12	AC	0	2.25	0	II
13-49	CN	Female	75	2.5	AC	0	2.5	0	II
11-107	AD	Male	71	4.32	AC	14	15	0	VI
11-27	AD	Male	89	2.2	AC	12.5	10	0	V
11-38	AD	Male	64	2.33	AC	14.5	15	0	VI
11-78	AD	Male	82	2.95	AC	14.5	15	0	V
13-10	AD	Male	74	2.58	AC	14	14	0	VI
12-27	AD	Female	89	2.7	AC	15	15	0	VI

* Frozen brain samples were obtained from Banner Sun Health Institute, Sun City Arizona [30]; CN = cognitively normal and may include mild-cognitive-impairment (MCI) subjects; AD = Alzheimer’s disease; PMI: postmortem interval in hours; LB = Lewy bodies. Plaque total: Includes neuritic, cored, and diffuse in the frontal, temporal, parietal, hippocampal, and entorhinal cortices. Semi-quantitative scores of none, sparse, moderate, and frequent were converted to numerical values of 0–3 for each region and summed to provide the plaque total. Tangle total: neurofibrillary tangle density in frontal, temporal, and parietal lobes; hippocampal CA1 region; and entorhinal cortical regions. Numerical values 0–3 for each region were summed to provide the tangle total. Braak score: Braak neurofibrillary stage (0–VI) defined in [11,36]. ^1^ AC: anterior cingulate containing corpus callosum; ^2^ Microscopic changes of AD; insufficient for AD diagnosis. Brain slices (10 μm thickness) were obtained from the chunks of frozen tissue on a Leica 1850 cryotome cooled to −20 °C and collected on Fisher slides.

It should be noted that subject CN 12-21 was found to exhibit more [^18^F]FAZIN3 binding compared to other CN subjects, which may be due to the presence of abundant Aβ plaques (Table 1). This subject was not confirmed to have AD. The exclusion of CN 12-21 from the CN group would further increase the percent difference between the AD and CN groups.

In order to further ascertain this increase in MAO-A activity in the six AD subjects, we prepared [^18^F]FEH, a known fluorine-18 analog of [^11^C]harmine, a MAO-A radiotracer [28,29]. All AD and CN subjects exhibited preferential binding in the anterior cingulate (GM) regions. Increased binding of [^18^F]FEH was observed in AD brains compared to that in CN brains (Supplementary Figure S1). The average [^18^F]FEH AD GM/WM ratio = 1.95, while the average CN GM/WM ratio = 1.50.

**Figure 3:**
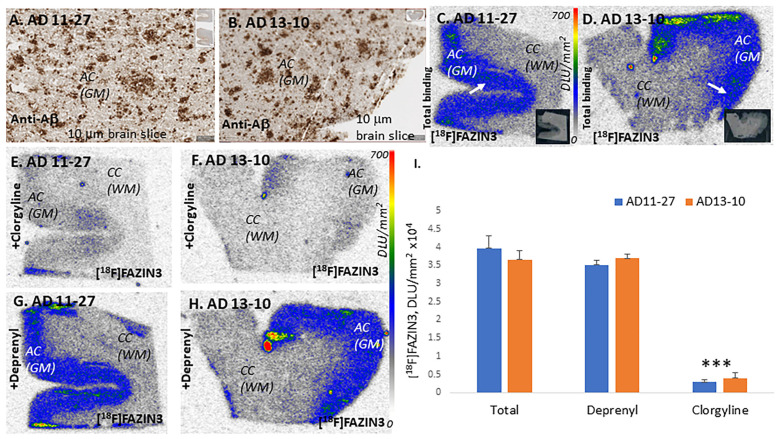
MAO drug effects on [^18^F]FAZIN3: (**A**,**B**). Anti-Aβ AD 11-27 and AD 13-10, 10 μm brain slice showing abundant Aβ plaques in anterior cingulate (GM). (**C**,**D**). MAO-A in GM labeled by [^18^F]FAZIN3 in brain sections of AD 11-27 and AD 13-10 (arrows indicate Aβ plaques in (**A**,**B**); inset shows scan of the same brain slices). (**E**,**F**). MAO-A drug clorgyline, 1 μM, displaced >90% [^18^F]FAZIN3 in adjacent sections of AD 11-27 and AD 13-10. (**G**,**H**). MAO-B drug (*R*)-deprenyl, 1 μM, had little effect on [^18^F]FAZIN3 binding in of AD 11-27 and AD 13-10. (**I**). Plot of [^18^F]FAZIN3 total binding, in the presence of (*R*)-deprenyl and clorgyline in AD subjects showing no effect of (*R*)-deprenyl while clorgyline displaced >90% of [^18^F]FAZIN3 (“*** = *p* < 0.001” for total versus clorgyline; total versus (*R*)deprenyl was not significant. Autoradiography scale bar: 0 to 700 DLU/mm^2^.

Selective binding of [^18^F]FAZIN3 to MAO-A is shown in Figure 3A–F. Two AD subjects (11-27 and 13-10) are shown to have abundant Aβ plaques (Figure 3A,B). Significant levels of [^18^F]FAZIN3 binding are observed in the GM of both the subjects (Figure 3C,D), which is also shown in Figure 3I for both the subjects. In the presence of the irreversible MAO-A inhibitor clorgyline at a concentration of 1 μM, over 90% of [^18^F]FAZIN3 binding was displaced in the AD subjects (Figure 3E,F). On the other hand, Figure 3G,H shows little effect of the irreversible MAO-B inhibitor (*R*)-deprenyl on [^18^F]FAZIN3 binding, suggesting [^18^F]FAZIN3 as a selective MAO-A radiotracer. Figure 3I shows the extent of the MAO drug effects on [^18^F]FAZIN3 binding. All CN subjects exhibited similar drug effects, further confirming that the observed binding of [^18^F]FAZIN3 is to MAO-A. This MAO-A selectivity of [^18^F]FAZIN3 was also reported previously [24].

### 2.2. Aβ Plaque Imaging

**Figure 4:**
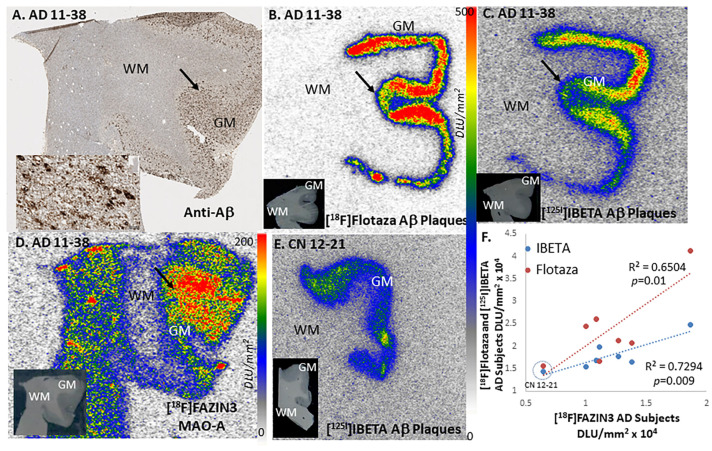
Aβ Plaque Imaging with [^18^F]Flotaza, [^125^I]IBETA, and MAO-A Imaging with [^18^F]FAZIN3: (**A**). AD brain slice of subject AD 11-38 showing anti-Aβ immunostained gray matter (GM) in the anterior cingulate and a lack of Aβ plaques in white matter (WM) in the corpus callosum (inset shows Aβ plaques at arrow at 50 µm); (**B**). [^18^F]Flotaza binding to Aβ plaques in the gray matter regions in adjacent slices of subject AD 11-38 (inset shows scan of the brain slice). (**C**). [^125^I]IBETA binding to Aβ plaques in the gray matter regions in adjacent slices of subject AD 11-38 (inset shows scan of the brain slice). (**D**). High levels of [^18^F]FAZIN3 binding in gray matter in adjacent slice of AD 11-38 subject showing binding to MAO-A in the GM regions (inset shows scan of the brain slice). (**E**). [^125^I]IBETA binding to Aβ plaques in the gray matter regions in CN subject CN 12-21 (inset shows scan of the brain slice). (**F**). Linear correlation plot of [^18^F]flotaza and [^125^I]IBETA with [^18^F]FAZIN3 binding in all 6 AD subjects including the one CN 12-21 subject (dotted circle) shows positive relationship (r^2^ =0.65, *p =* 0.01 with [^18^F]flotaza and r^2^ =0.73, *p =* 0.009 with [^125^I]IBETA). Autoradiography scale bar: 0 to 500 digital light units (DLU)/mm^2^ for (**B**,**C**) and (**E**) and 0 to 200 digital light units (DLU)/mm^2^ for (**D**).

[^18^F]Flotaza is a new fluorinated PEG derivative for Aβ plaque PET imaging and provides high-contrast imaging of postmortem AD brains [25]. All AD subjects showed high levels of [^18^F]flotaza binding in the anterior cingulate regions. Figure 4A shows AD subject 11-38, confirming the presence of extensive Aβ plaques in the anterior cingulate GM regions (shown in the inset). The adjacent brain slice for subject AD 11-38 showed consistent cortical binding of [^18^F]flotaza corresponding to anti-Aβ (Figure 4B). Further confirmation of Aβ plaques was achieved by [^125^I]IBETA, a new Aβ plaque imaging agent shown in Figure 4C [35]. Similar to the [^18^F]flotaza and [^125^I]IBETA binding, [^18^F]FAZIN3 exhibited binding across the cortical layers of the anterior cingulate (Figure 4D). Figure 4E shows the presence of Aβ plaques in one CN subject, CN 12-21, using [^125^I]IBETA. Linear correlations with r^2^ = 0.65 and r^2^ = 0.73 of the GM binding of [^18^F]FAZIN3 with [^18^F]flotaza and [^125^I]IBETA, respectively (Figure 4F), were seen in all AD subjects, including CN 12-21. The presence of Aβ plaques in CN 12-21 was also confirmed with [^18^F]flotaza (Supplementary Figure S2A). Both Aβ-plaque binding agents [^18^F]flotaza and [^125^I]IBETA exhibited a moderate to strong positive relationship with [^18^F]FAZIN3 binding to MAO-A (*p =* 0.01 and *p =* 0.009, respectively, Figure 4F).

### 2.3. Tau Imaging

**Figure 5:**
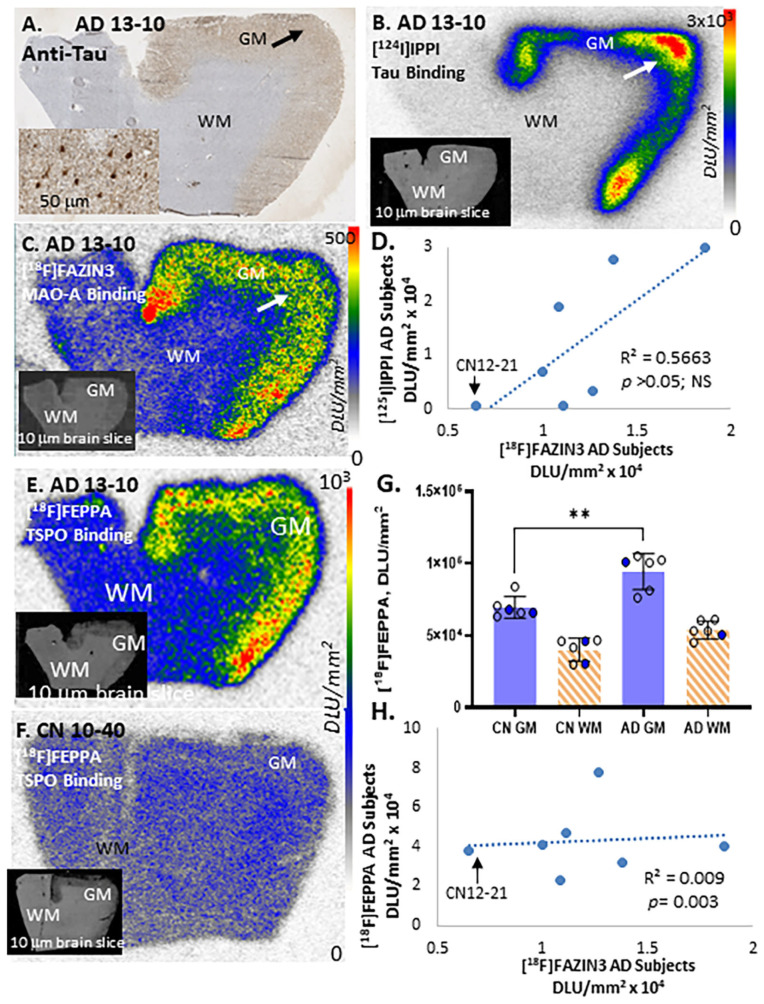
Tau Imaging with [^125^I]IPPI, TSPO Imaging with ^18^F]FEPPA, and MAO-A Imaging with [^18^F]FAZIN3: (**A**). Anti-tau immunohistochemical staining revealed a presence of NFT in the AD 13-10 subject. Inset at 50 µm magnification shows a presence of NFT in the GM (arrow). (**B**). [^124^I]IPPI binding to tau was observed in the gray matter of the adjacent slice of the AD 13-10 subject. Little nonspecific [^124^I]IPPI binding was seen in WM (inset shows scan of the brain slice). (**C**). Binding of [^18^F]FAZIN3 in gray matter in the adjacent slice of the AD 13-10 subject shows extensive binding to MAO-A in the GM regions (inset shows scan of the brain slice). (**D**). Linear regression plot of [^125^I]IPPI and [^18^F]FAZIN3 binding in all 6 AD subjects shows a modest positive correlation (r^2^ = 0.57) but not significant (*p* > 0.05). (**E**). Brain slices of AD subject 13-10 shows high binding of [^18^F]FEPPA to TSPO in the gray matter regions of the anterior cingulate (inset shows scan of the brain slice). (**F**). Lower levels of [^18^F]FEPPA binding are observed in the gray matter of the CN 10-40 subject (inset shows scan of the brain slice). (**G**). Average of [^18^F]FEPPA binding in the GM regions of 6 AD subjects was greater than that of the 6 CN subjects (** *p* < 0.01; open circles males, solid circles females). Ratio of AD GM-to-CN GM = 1.65, suggesting increased TSPO expression in the AD brains. (**H**). Linear plot of [^18^F]FEPPA and [^18^F]FAZIN3 binding in all 6 AD subjects exhibited significant positive correlation (*p* < 0.01). Autoradiography scale bar: 0 to 3000 digital light units (DLU)/mm^2^ for (**B**,**E**) and (**F**) and 0 to 500 digital light units (DLU)/mm^2^ for (**C**).

We developed [^125^I]IPPI [26], an analog of [^18^F]MK-6240, for selective binding to tau, which is useful in autoradiographic studies. More recently, [^124^I]IPPI was developed as a potential in vivo PET imaging agent for tau [37]. Figure 5A shows anti-tau in AD subject 13-10, confirming the presence of NFT in the anterior cingulate GM regions (shown in the inset and arrow). Figure 5B shows the adjacent brain slice for subject AD 13-10 with binding of [^124^I]IPPI in the anterior cingulate regions rich in NFT. The GM regions showed significantly higher binding compared to that in the WM (Figure 5B). All AD subjects showed high levels of [^125^I]IPPI binding in the anterior cingulate regions, although there was greater variability in the level of binding compared to that in [^18^F]flotaza and [^125^I]IBETA for Aβ plaques. As expected, CN subjects did not have any [^125^I]IPPI binding in the GM regions (Supplementary Figure S2). The adjacent section of AD subject 13-10 shows [^18^F]FAZIN3 binding across the cortical layers of the anterior cingulate (Figure 5C). A positive correlation (r^2^ = 0.49) of the GM binding of [^125^I]IPPI and [^18^F]FAZIN3 was found in all AD subjects but was not significant (Figure 5D). Spearman’s correlation coefficient was also not found to be significant.

### 2.4. TSPO Imaging

Shown in Figure 5E is the brain slice of AD subject 13-10, showing high binding of [^18^F]FEPPA to the TSPO in the gray matter regions of the anterior cingulate. Nonspecific binding of [^18^F]FEPPA was observed in the WM. Lower levels of [^18^F]FEPPA binding were observed in CN subject 10-40 (Figure 5F). The binding of [^18^F]FEPPA from the anterior cingulate was displaced by using PK 11195 (10 μM; [23]). Because of the high levels of specific binding of [^18^F]FEPPA in all the subjects, it may be assumed that the subjects are high-affinity binders. All CN subjects exhibited lower [^18^F]FEPPA binding in the GM (Figure 5G) compared to the AD subjects. The increased binding of [^18^F]FEPPA in the GM of the AD subjects versus that in the CN subjects was significant (*p* < 0.01; Figure 5G). The average GM/WM ratio was 1.85 for the AD subjects and 1.72 for the CN subjects, suggesting an approximate 11% increase in AD subjects. The ratio of [^18^F]FEPPA binding in GM alone of the 6 AD subjects and 6 CN subjects was found to be 1.65. The specific binding of [^18^F]FEPPA and [^18^F]FAZIN3 exhibited a significant, low positive correlation in the AD subjects, as well as in CN 12-21 (Figure 5H).

**Figure 6:**
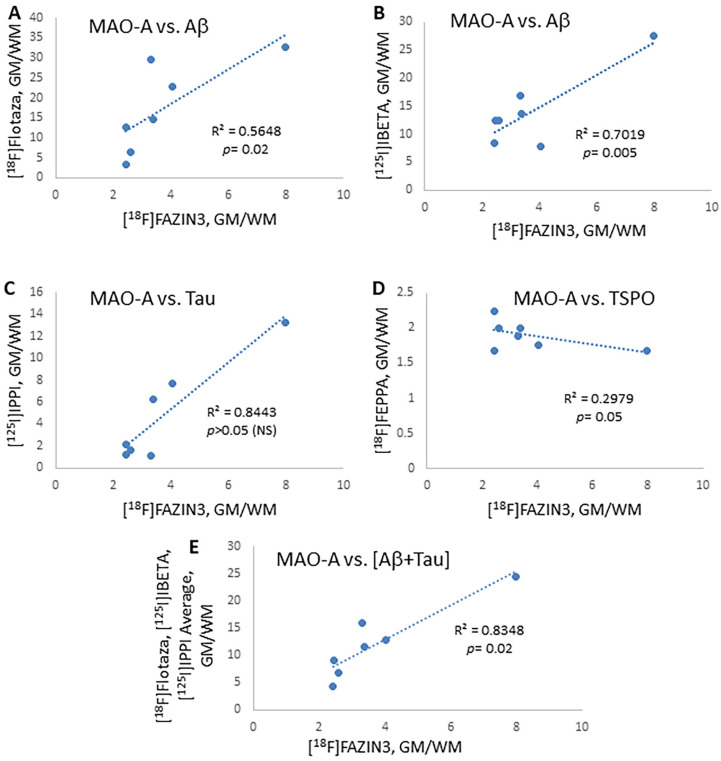
Correlation Plots of GM/WM Ratios of [^18^F]FAZIN3 with other Biomarkers: (**A**). GM/WM ratio of [^18^F]FAZIN3 binding to MAO-A in all the six AD subjects and one CN subject (with positive Aβ) showed a weak positive correlation (r^2^ = 0.56; *p* = 0.018) with the [^18^F]flotaza GM/WM binding ratio to Aβ plaques. (**B**). A better positive correlation (r^2^ = 0.70; *p =* 0.005) of the [^18^F]FAZIN3 GM/WM ratio was observed with the [^125^I]IBETA GM/WM binding ratio to Aβ plaques. (**C**). A good correlation (r^2^ = 0.84; *p* > 0.05, NS) of the [^18^F]FAZIN3 GM/WM ratio was observed with the [^125^I]IPPI GM/WM binding ratio to tau. (**D**). Binding of the [^18^F]FAZIN3 GM/WM ratio did not show a positive correlation with the [^18^F]FEPPA GM/WM binding ratio to TSPO. The trend appeared to be a negative correlation (r^2^ = 0.30; *p* = 0.05). (**E**). A plot of [^18^F]flotaza, [^125^I]IBETA, and [^125^I]IPPI averages versus the [^18^F]FAZIN3 GM/WM ratio provides a good linear regression with high significance (r^2^ = 0.83; *p =* 0.02).

The correlation of the GM/WM binding ratios of the four individual biomarkers in the 6 AD subjects and one CN subject is shown in Figure 6. A positive correlation of MAO-A with Aβ plaque levels was observed with both [^18^F]flotaza (Figure 6A) and [^125^I]IBETA (Figure 6B) and was found to be significant (*p* = 0.02 and *p* = 0.005, respectively). A stronger MAO-A-to-tau linear regression was seen with the GM/WM ratios of [^125^I]IPPI (Figure 6C), compared to when only GM binding was used (Figure 5D). However, in both cases, the correlation between [^125^I]IPPI and [^18^F]FAZIN3 was not found to be significant. In the case of TSPO, labeled with [^18^F]FEPPA, there was a lower but significant correlation that appeared negative (Figure 6D). When Aβ plaque and tau were correlated together with [^18^F]FAZIN3, a significant stronger correlation was observed (*p =* 0.02; Figure 6E). This positive correlation of MAO-A to both Aβ plaque and tau suggests that at least in the anterior cingulate cortex, in this limited number of subjects, the three biomarkers may be positively correlated. However, using one-way ANOVA multiple comparisons, the correlation of MAO-A levels to Aβ plaque levels was highly significant ([^18^F]FAZIN3 versus [^18^F]flotaza, *p =* 0.001 and [^18^F]FAZIN3 versus [^125^I]IBETA, *p =* 0.01), whereas [^18^F]FAZIN3 versus [^125^I]IPPI and [^18^F]FEPPA were not significant.

**Figure 7:**
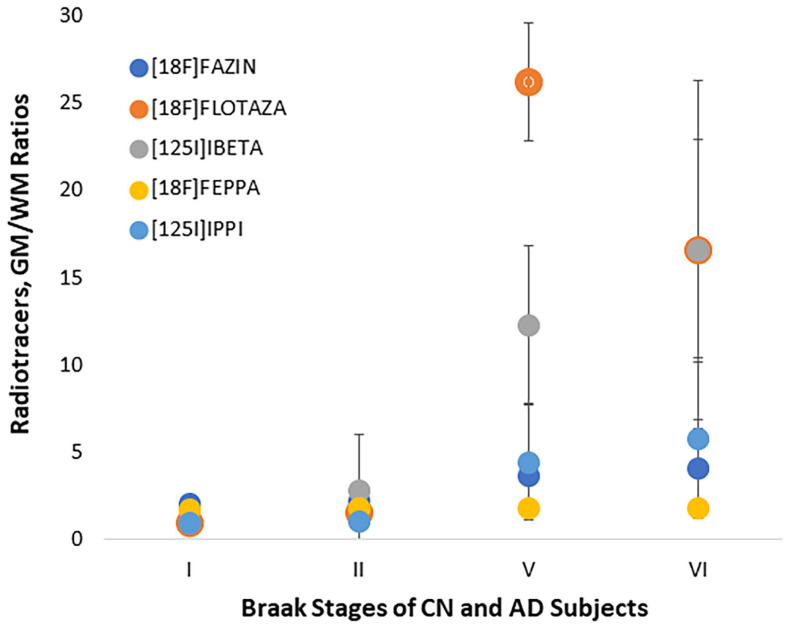
Radiotracer Binding to Different Braak Stages: Ratios of GM/WM of [^18^F]FAZIN (for MAO-A), [^18^F]flotaza (for Aβ plaques), [^125^I]IBETA (for Aβ plaques), [^18^F]FEPPA (for TSPO), and [^125^I]IPPI (for tau) in CN and AD subjects at Braak I, Braak II, Braak V, and Braak VI are shown.

Shown in Figure 7 is the comparison of the binding (GM/WM) of the five radiotracers with respect to the Braak stages. As expected, the Aβ and tau radiotracers did not exhibit any binding in Braak stages I and II, except for subject CN12-21, who exhibited significant presence of Aβ plaques but no tau (Supplementary Figure S2). An increase in Aβ is seen in Braak stages V and VI with both [^18^F]flotaza and [^125^I]IBETA, with GM/WM ratios >15 at Braak stage VI. Similarly, increases in tau were seen in Braak stages V and VI with [^125^I]IPPI GM/WM ratios >5 in Braak stage VI. There was greater variability in the presence of tau compared to that of the presence Aβ within each of these advanced stages. In the case of [^18^F]FAZIN3, there was a gradual increase in the GM/WM ratios, with the highest being in Braak stage VI. [^18^F]FEPPA exhibited the least change in its GM/WM ratio at the different stages.

## 3. Discussion

Studies in patients suffering from depression found increased levels of MAO-A radiotracer [^11^C]harmine in the anterior cingulate and temporal cortex [38]. Our recent findings in the postmortem PD anterior cingulate using [^18^F]FAZIN3 found significant increases in MAO-A binding [24]. No imaging studies on the status of MAO-A in AD have been reported. Since the cingulate cortex is amongst one of the brain regions affected by both Aβ plaques and NFT [31,32], this study evaluated the anterior cingulate in postmortem AD subjects. Additionally, previous PET imaging reports using the MAO-A radiotracer [^11^C]harmine confirmed significant levels of MAO-A in the anterior cingulate of healthy human subjects [30], and elevated levels of [^11^C]harmine binding to MAO-A were found in the anterior cingulate in major depression [38].

At least a 59% increase in [^18^F]FAZIN3 in AD subjects was observed when comparing ratios of GM/WM in AD and CN subjects. This increase in [^18^F]FAZIN3 binding was very significant and greater than that reported in depressed patients [38]. This is indicative of an increase in MAO-A in the anterior cingulate cortex in AD (either more MAO-A per mitochondrion or more mitochondria or more dysphoric mitochondria in AD). It should be noted that depression was not a comorbidity in the AD subjects. Based on the selectivity of [^18^F]FAZIN3 in AD subjects (Figure 3) and our previous finding in PD subjects [24], this study confirms that the increases reported here are MAO-A and not MAO-B. Although this is a small study and will need a larger patient sample, our preliminary findings suggest that a 59% increase in [^18^F]FAZIN3 binding compared to that of the control subjects in a postmortem study may be a sufficient increase to detect changes in MAO-A levels in AD. This will depend, however, on the need to successfully translate the use of [^18^F]FAZIN3 to in vivo human PET studies for the evaluation of MAO-A in AD.

Increases in [^18^F]FAZIN3 binding to MAO-A in AD subjects positively correlated with Aβ plaques labeled with [^18^F]flotaza [25] and [^125^I]IBETA [35]. It is noteworthy that one CN subject (CN 12-21) with significant Aβ plaques (Figure 4E) also had higher levels of [^18^F]FAZIN3 binding compared to the rest of the CN subjects without Aβ plaques (Figure 2I and Supplementary Figure S2). Since the formation and accumulation of Aβ plaques causes an inflammatory response [39], it is likely that cellular pathways trigger an increase in MAO-A levels [40,41]. This increase in MAO-A levels potentially may influence several detrimental effects, including depleting neurotransmitter levels and inducing further neurodegeneration from oxidative processes. It may be noted that the selective serotonin reuptake inhibitor fluoxetine, a common antidepressant, has been shown to accumulate in the mitochondria in micromolar concentrations and may be contributing to secondary mechanisms for its antidepressant effects [42,43,44].

The presence and spread of NFT in AD is currently being actively pursued as a more accurate biomarker for the staging of the disease [45]. All AD subjects in this study had significant levels of [^125^I]IPPI binding to tau, while the CN subjects had none. Although there was greater variance in the binding of [^125^I]IPPI in the AD subjects compared to that in the [^18^F]flotaza binding, there was a weak positive correlation (r^2^ = 0.49) of [^125^I]IPPI with [^18^F]FAZIN3, but it was not significant. For extended PET imaging, iodine-124 IPPI may serve as a PET imaging for tau (iodine 124 T_1/2_ = 4.2 days), similar to our efforts on extended imaging with iodine-124-labeled epidepride and Aβ plaques with [^124^I]IBETA and [^124^I]IAZA [35,38,46,47].

The inflammatory response in AD may include changes in the microglia morphology—from ramified (resting) to amoeboid (active)—and astrogliosis (manifested by an increase in the number, size, and motility of astrocytes) surrounding the senile plaques [48]. Microglia activation, a biomarker for inflammation, is characterized by an increased expression of TSPO. Postmortem studies and PET studies of AD have shown significantly elevated TSPO expression in several brain regions [49]. [^18^F]FEPPA, which has a high affinity for TSPO, has shown promise in PET studies of AD [50]. In the present study, although the AD subjects had a marginal increase in [^18^F]FEPPA binding compared to the CN subjects, there was a low correlation with [^18^F]FAZIN3 binding. In contrast, for MAO-B, there appeared to be a better correlation between deprenyl and PK11195 [23]. Since both MAO-B and TSPO are located in glial cells, a stronger correlation of the two biomarkers may be expected. On the other hand, the distribution of MAO-A is different and may be predominantly presynaptic, which could account for the poor correlation of [^18^F]FAZIN3 binding to MAO-A with [^18^F]FEPPA binding to TSPO. However, these findings are preliminary, and a larger study will be needed to ascertain this. Additional biomarkers for neuroinflammation will have to be explored for potential correlations with changes in MAO-A in AD [40,41].

Our results suggest that increases in MAO-A levels appear to strongly correlate with Aβ plaques and may serve as a complimentary biomarker for AD. The presence of NFT with SP may further increase the levels of MAO-A. Using measurements of the enzymatic activity of MAO-A in the prefrontal cortex, it has been suggested that changes in MAO-A and B levels occur early in AD and remain at the same levels with increasing duration of the disease [18]. Mitochondrial dysfunction appears to affect both MAO-B and MAO-A [51]. Their potential involvement in the continued accumulation and increase in both SP and NFT loads as well as in the depletion of neurotransmitter function in the AD brain needs further studies. Our assessment in this preliminary study suggests that significant increases in MAO-A levels occur alongside the formation of Aβ plaques and may continue with the formation of NFT. A direct role of TSPO in increasing MAO-A levels was not found in this study.

This is an initial imaging study demonstrating that levels of MAO-A in the AD brain are altered. Limitations of the study include the small number of subjects in advanced stages of AD. A larger study with more subjects at different disease stages is needed to ascertain the correlation of MAO-A changes with the progression of disease. Our study here reports only one brain region; other brain regions, such as the temporal cortex and hippocampus, need to be studied to assess MAO-A changes. It must also be noted that depressive behaviors are often present in AD [52], although our cohort of subjects in this study were not diagnosed with depression. Since MAO-A appears to be upregulated in depression [38], our future studies will also have to evaluate earlier, clinically asymptomatic cases of AD. Finally, attempts are also underway to optimize immunohistochemical staining methods for MAO-A in adjacent brain slices so that the binding of [^18^F]FAZIN3 may be compared.

## 4. Materials and Methods

### 4.1. General Methods

Iodine-125 sodium iodide was purchased from American Radiolabeled Chemicals, Inc., St. Louis, MO, USA; iodine-124 sodium iodide was purchased from 3D Imaging, Little Rock, AR; and fluorine-18 was purchased from PETNET, Inc. Specialty chemicals MK-6240 and PK 11195 were purchased from AbaChemScene, New Jersey; and clorgyline and (*R*)-deprenyl were purchased from Research Biochemicals (Sigma Aldrich, St. Loius, MO, USA). FEPPA tosylate precursor and PIB (Pittsburgh compound B) were purchased from ABX Inc., Radeberg, Germany. 

### 4.2. Postmortem Human Brain

Human postmortem brain tissue samples were obtained from Banner Sun Health Research Institute (BHRI), Sun City, AZ, USA, brain tissue repository for in vitro experiments. Well-characterized frozen brain samples were obtained from BHRI, Sun City Arizona (Table 1; [53]). Brain tissue samples from AD and cognitively normal (CN) subjects were selected by observing the presence and absence of end-stage pathology. The brain slices contained the anterior cingulate and corpus callosum regions (CN, *n* = 6; ages 81–90 and AD, *n* = 6, ages 64–89; Table 1). Brain sections were stored at −80 °C. All postmortem human brain studies were approved by the Institutional Biosafety Committee of University of California, Irvine.

### 4.3. Autoradiography

Brain slices were placed in separate incubation chambers and were allowed to thaw from −80 °C to ambient temperature for 10–15 min. Subsequently, they were preincubated in PBS (pH 7.4) or Tris buffer (pH 7.4) at ambient temperature for 10 min. Fresh PBS buffer (pH 7.4) or Tris buffer (pH 7.4) containing the respective radiotracer was added to all the chambers and incubated for 60–90 min. The brain sections were air-dried, exposed (24 h to 7 days, depending on radioisotope) on phosphor screens, and then placed on the Phosphor Autoradiographic Imaging System (Packard Instruments Co., Boston, MA, USA). Using the Optiquant acquisition and analysis program (Packard Instruments Co., Boston, MA, USA), regions of interest were drawn in the gray matter regions of the anterior cingulate and white matter regions of the corpus callosum. Digital light units/mm^2^ (DLU/mm^2^) were used to quantify the extent of binding. Slides were scanned on a Hewlett Packard scanner to help delineate regions of the brain sections more clearly. At least *n* = 3 to 6 tissue sections per subject, per radiotracer were used for the study.

### 4.4. MAO-A Imaging

The azaindole derivative [18F]FAZIN3 was prepared in-house, as described previously [24]. Human brain slices containing the anterior cingulate and corpus callosum (10 μm thick) were placed in glass chambers and preincubated in PBS buffer for 10 min. The brain sections (CN and AD) were then incubated with [18F]FAZIN3 (approximately 120 kBq/mL; 1 nM; specific activity >35 GBq/μmol) in PBS at 25 °C for 1 h. The slices were then washed with cold PBS buffer (2 × 5 min) and rinsed with cold deionized water for 2 min. Drug competition studies using 1 μM clorgyline for MAO-A and 1 μM (R)-deprenyl for MAO-B were carried out on all CN and AD brain sections [26]. The brain sections were air-dried and exposed overnight on phosphor screens.

[18F]Fluoroethyl harmol ([18F]FEH) was produced in-house, as reported [28,29]. [18F]FEH was in 10% ethanol in sterile saline for in vitro studies. Human brain slices containing the anterior cingulate and corpus callosum (10 μm thick) were placed in a glass chamber and preincubated in PBS buffer for 10 min. The brain sections were placed in a glass chamber and incubated with [18F]FEH (approximately 37 kBq/mL; 1 nM; specific activity >35 GBq/μmol) in PBS at 25 °C for 1 h. The slices were then washed with cold buffer (2 × 5 min) and rinsed with cold deionized water for 2 min. The binding of [18F]FEH to MAO-A was confirmed by the blocking effects of 10 μM clorgyline. The brain sections were air dried and then exposed overnight on a phosphor film.

### 4.5. Aβ Plaque Imaging

Purified [18F]flotaza and [125I]IBETA were used for autoradiographic studies [25]. Human brain sections (10 μm thick) were placed in a glass chamber and preincubated in PBS buffer for 10–15 min. The brain sections were placed in glass chambers and incubated with [18F]flotaza (approximately 74–111 kBq/mL; 0.5–0.75 nM; specific activity >35 GBq/μmol)) in 40% ethanol-PBS buffer at 25 °C for 1.5 h. The slices were then washed with cold PBS buffer (1 × 5 min), 60% ethanol-PBS buffer (2 × 5 min), PBS buffer (1 × 5 min), and cold deionized water (2 min), respectively. Nonspecific binding was measured in the presence of 10 μM PIB. The brain sections were air-dried and then exposed overnight on phosphor screens. Aβ plaques were also imaged using our recently developed [125I]IBETA (60 mL; 3.7 kBq/mL; 0.2–0.5 nM; specific activity >90 GBq/μmol) using a similar procedure to that described above [34].

### 4.6. Tau Imaging

For tau imaging, [125I]IPPI was used for autoradiographic studies [26]. Human brain tissues from the 6 AD and 6 CN subjects were preincubated in PBS buffer for 15 min. After the preincubation buffer was discarded, [125I]IPPI in 10% ethanol PBS buffer with pH 7.4 (60 mL; 3.7 kBq/mL; 0.2–0.5 nM; specific activity >90 GBq/μmol) or [124I]IPPI (6 kBq/mL; 0.2-0.5 nM; specific activity >200 GBq/μmol)) [37] were added to the chambers and incubated at 25 °C for 1.25 h. Nonspecific binding was measured in separate chambers in the presence of 1 μM MK-6240. The slices were then washed with cold PBS buffer for 2 min, 50% ethanolic PBS buffer twice for 2 min each, PBS buffer for 2 min, and cold water for 1 min, respectively. The brain sections were air-dried and then exposed for a week on a phosphor film.

### 4.7. TSPO Imaging

The TSPO PET probe [18F]FEPPA, with >95% radiochemical purity and a specific activity of >70 GBq/μmol (>2 Ci/μmol), was used for autoradiographic studies [54]. Human brain slices from all 6 CN and 6 AD subjects were placed in glass chambers and preincubated in 0.1 M Tris buffer (pH 7.4) for 10 min. Following preincubation, fresh buffer was added to the chambers along with [18F]FEPPA (approximately 37–50 kBq/mL; 0.5–0.75 nM; specific activity >15 GBq/μmol) in 0.1 M Tris buffer, pH 7.4 at 25 °C, and incubated for 1 h. The slices were then washed with cold Tris buffer (2 × 5 min) and rinsed with cold deionized water for 2 min. Nonspecific binding was measured in the presence of 10 μM PK 11195. The brain sections were air-dried and then exposed overnight on phosphor screens.

### 4.8. Immunohistochemistry

Immunostaining of all brain sections was carried out by University of California-Irvine, Pathology services using Ventana BenchMark Ultra protocols. Neighboring slices were immunostained with DAKO polyclonal total tau antibody which detects all 6 six isoforms of tau, dilution 1: 3000, A0024 (Agilent, CA, USA) using reported protocols [55]. For Aβ plaques, slices from all subjects were immunostained with anti-Aβ Biolegend 803015 (Biolegend, CA, USA), which is reactive to amino acid residue 1-16 of β-amyloid [56]. All IHC-stained slides were scanned using the Ventana Roche instrumentation and analyzed using QuPath [37,57].

### 4.9. Image Analysis

All regions of interest (ROI) in the anterior cingulate (GM) and corpus callosum (WM) autoradiographic images of [^18^F]FAZIN3, [^18^F]flotaza, [^125^I]IPPI, [^18^F]FEPPA, and [^18^F]FEH were quantified using measurements (DLU/mm^2^). Immunostained sections were analyzed using QuPath. Gray matter (GM) and WM binding of each radiotracer in the AD and CN subjects were measured, and the GM/WM ratios of the AD and CN subjects were compared for each radiotracer. Using the ratio method is akin to in vivo PET methods, where the standard uptake value (SUV) between the target region is compared to a nonspecific binding reference region as a ratio and expressed as SUVR [58,59]. Group differences of GM/WM ratios between AD and CN subjects were evaluated using *t*-tests. For each radiotracer, the specific binding was calculated by subtracting the WM from the GM of each individual subject. The specific binding of [^18^F]FAZIN3 was correlated to each specific binding of [^18^F]flotaza, [^125^I]IPPI, and [^18^F]FEPPA in order to assess any relationship between the different biomarkers.

### 4.10. Statistical Analysis

Group differences between AD and CN subjects were assessed using the average GM/WM ratios and were determined using Microsoft Excel 16 and GraphPad Prism 9. The statistical power was determined with Student’s *t*-test, and *p* values of <0.05 were considered to indicate statistical significance. Spearman’s correlation was carried out in certain cases. The linear correlations and ANOVA analysis of the bindings between the different radiotracers were used to evaluate potential relationships between the different biomarkers.

## 5. Conclusions

Four different biomarkers, which included Aβ plaques, tau, the translocator protein for microglia, and MAO-A, were used to study the anterior cingulate cortex of well-characterized AD subjects. Our results showed that significantly greater MAO-A activity was present in the anterior cingulate of AD subjects compared to the cognitively normal controls. This increased MAO-A in AD subjects was positively correlated to Aβ plaque and tau levels. Early assessment of increased MAO-A levels using PET imaging in AD patients may provide opportunities for therapeutic interventions by MAO drugs [60]. This may provide neuroprotection and slow down AD progression. Increased MAO-A levels did not correlate with the expression of the translocator protein. A larger study with more subjects at different disease stages is needed in order to ascertain the correlation of MAO-A imaging with Aβ plaque and tau levels. Other brain regions such as the temporal cortex and hippocampus need to be studied to assess MAO-A increases.

## Data Availability

The data that supports the findings of this study are available from the corresponding author for discussions upon reasonable requests.

## Supplementary Materials

The supporting information can be downloaded at: https://www.mdpi.com/article/10.3390/ijms241310808/s1.
