# Supplementary material for: Comparison of Monoamine Oxidase-A, Aβ Plaques, Tau, and Translocator Protein Levels in Postmortem Human Alzheimer’s Disease Brain
Source: Int J Mol Sci. 2023 Jun 28;24(13):10808. doi: 10.3390/ijms241310808 (PMC10341404; doi:10.3390/ijms241310808)
Supplement: Supplementary file 1 [file ijms-24-10808-s001.zip › ijms-2435327-supplementary.pdf]

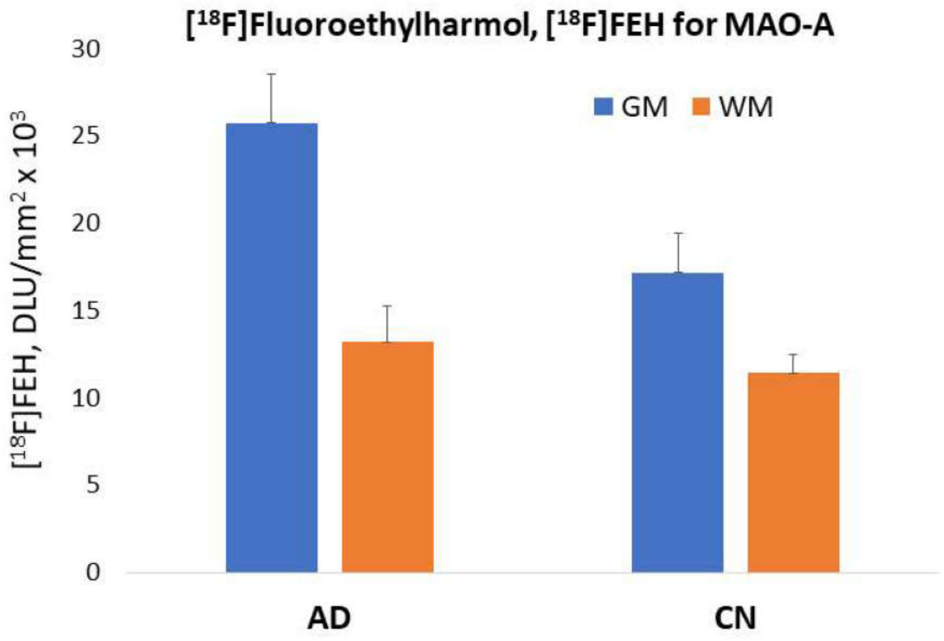

Figure S1. [18F]Fluoroethylharmol ([18F]FEH): [18F]FEH, a fluorinated analog of harmine has been developed as a PET imaging agent for MAO-A (Maschauer et al., 2015). We used [18F]FEH to examine binding to the AD and CN brain sections. [18F]FEH binding was higher in the anterior cingulate (grey matter, GM) of AD subjects compared to controls as seen in figure below ($p < 0.05$). However, white matter (WM) binding in corpus callosum was significant, thus reducing the ratio of GM to WM. The GM/WM ratio in AD subjects was 1.95 whereas in the CN subjects it was 1.50. Thus, AD subjects had a 30% increase in [18F]FEH binding.

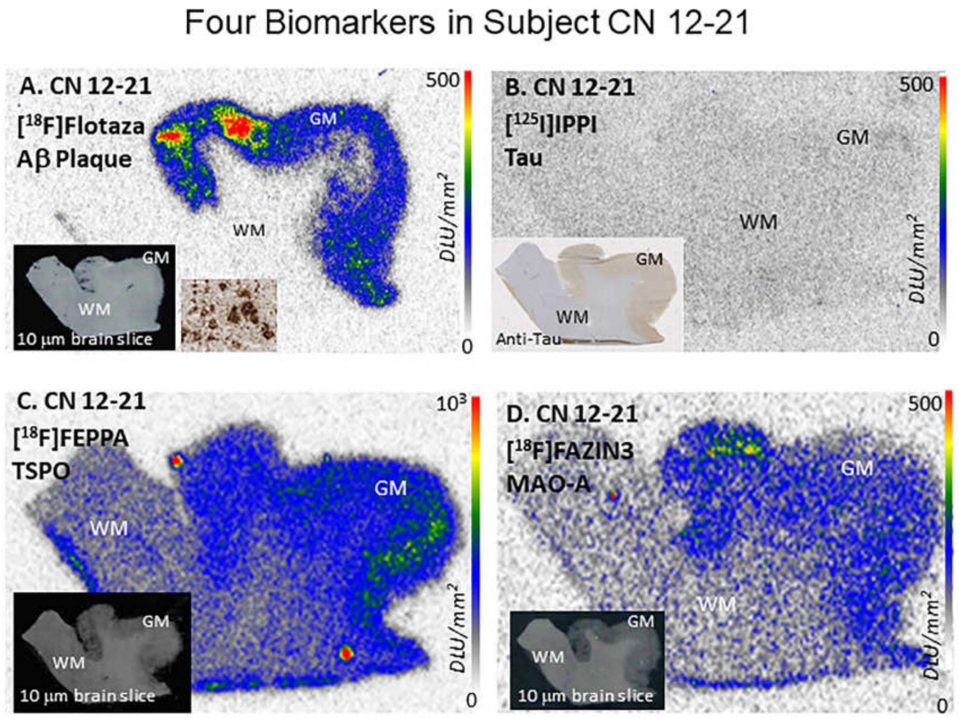

Figure S2. Four Biomarkers in Subject CN 12-21: Four biomarkers in cognitively normal CN 12-21 subject with microscopic changes of AD but insufficient for AD diagnosis. (A). Aβ amyloid plaques were observed with [^{18}F]flotaza (inset shows brain slice and anti Aβ plaque staining) and consistent with findings of [^{125}I]IBETA shown in Figure 4E, (B). [^{125}I]IPPI was used to evaluate Tau and images ([^{125}I]IPPI and anti-Tau in inset) show absence of Tau in CN 12-21. (C). [^{18}F]FEPPA was used for TSPO with gray matter (GM) showing higher levels than white matter (WM). (D). Monoamine oxidase A (MAO-A) levels in CN 12-21 measured using [^{18}F]FAZIN3 was found to be higher compared to other CN subjects as shown in Figure 2I.
